# Supplementary material for: SE(3)-equivariant ternary complex prediction towards target protein degradation
Source: Nat Commun. 2025 Jul 1;16:5514. doi: 10.1038/s41467-025-61272-5 (PMC12216337; doi:10.1038/s41467-025-61272-5)
Supplement: Supplementary file 2 — Reporting Summary [file 41467_2025_61272_MOESM2_ESM.pdf]

nature portfolio

Corresponding author(s): Wenbing Huang, Yi Yang, and Weixian Deng

Last updated by author(s): May 26, 2025

Reporting Summary

Nature Portfolio wishes to improve the reproducibility of the work that we publish. This form provides structure for consistency and transparency in reporting. For further information on Nature Portfolio policies, see our Editorial Policies and the Editorial Policy Checklist.

Statistics

For all statistical analyses, confirm that the following items are present in the figure legend, table legend, main text, or Methods section.

n/a	Confirmed	
[ ]	[x]	The exact sample size (*n*) for each experimental group/condition, given as a discrete number and unit of measurement
[ ]	[x]	A statement on whether measurements were taken from distinct samples or whether the same sample was measured repeatedly
[ ]	[x]	The statistical test(s) used AND whether they are one- or two-sided<br>*Only common tests should be described solely by name; describe more complex techniques in the Methods section.*
[x]	[ ]	A description of all covariates tested
[x]	[ ]	A description of any assumptions or corrections, such as tests of normality and adjustment for multiple comparisons
[ ]	[x]	A full description of the statistical parameters including central tendency (e.g. means) or other basic estimates (e.g. regression coefficient) AND variation (e.g. standard deviation) or associated estimates of uncertainty (e.g. confidence intervals)
[ ]	[x]	For null hypothesis testing, the test statistic (e.g. *F*, *t*, *r*) with confidence intervals, effect sizes, degrees of freedom and *P* value noted<br>*Give P values as exact values whenever suitable.*
[x]	[ ]	For Bayesian analysis, information on the choice of priors and Markov chain Monte Carlo settings
[x]	[ ]	For hierarchical and complex designs, identification of the appropriate level for tests and full reporting of outcomes
[x]	[ ]	Estimates of effect sizes (e.g. Cohen's *d*, Pearson's *r*), indicating how they were calculated

Our web collection on statistics for biologists contains articles on many of the points above.

Software and code

Policy information about availability of computer code

Data collection	No software was used for data collection.
Data analysis	Python3, ChimeraX 1.7, BioPython 1.81, MMsequence2 were used to analyze the data. The code is available at https://github.com/youqingxiaozhua/DeepTernary.

For manuscripts utilizing custom algorithms or software that are central to the research but not yet described in published literature, software must be made available to editors and reviewers. We strongly encourage code deposition in a community repository (e.g. GitHub). See the Nature Portfolio guidelines for submitting code & software for further information.

Data

Policy information about availability of data

All manuscripts must include a data availability statement. This statement should provide the following information, where applicable:

- Accession codes, unique identifiers, or web links for publicly available datasets
- A description of any restrictions on data availability
- For clinical datasets or third party data, please ensure that the statement adheres to our policy

The TernaryDB complex list, cluster results of the training dataset, training log, pre-trained model weights, and unbound structures for PROTAC testing have been deposited at https://github.com/youqingxiaozhua/DeepTernary for public access. The complex crystal structures are downloaded from the Protein Data Bank at https://www.rcsb.org/. Source Data are provided with this paper. The PDB IDs analysed in this manuscript are: 5T35 [http://doi.org/10.2210/pdb5T35/pdb], 6W7O [http://doi.org/10.2210/pdb6W7O/pdb], 6W8I [http://doi.org/10.2210/pdb6W8I/pdb], 7BQU [http://doi.org/10.2210/pdb7BQU/pdb], 4JDD [http://

doi.org/10.2210/pdb4JDD/pdb], 4TR9 [http://doi.org/10.2210/pdb4TR9/pdb], 6HAX [http://doi.org/10.2210/pdb6HAX/pdb], and 6BN7 [http://doi.org/10.2210/pdb6BN7/pdb].

Research involving human participants, their data, or biological material

Policy information about studies with human participants or human data. See also policy information about sex, gender (identity/presentation), and sexual orientation and race, ethnicity and racism.

Reporting on sex and gender	Not applicable to this work
Reporting on race, ethnicity, or other socially relevant groupings	Not applicable to this work
Population characteristics	Not applicable to this work
Recruitment	Not applicable to this work
Ethics oversight	Not applicable to this work

Note that full information on the approval of the study protocol must also be provided in the manuscript.

Field-specific reporting

Please select the one below that is the best fit for your research. If you are not sure, read the appropriate sections before making your selection.

[x] Life sciences [ ] Behavioural & social sciences [ ] Ecological, evolutionary & environmental sciences

For a reference copy of the document with all sections, see nature.com/documents/nr-reporting-summary-flat.pdf

Life sciences study design

All studies must disclose on these points even when the disclosure is negative.

Sample size	The dataset utilized for training consists of totally 25,756 ternary complexes. To ensure an unbiased evaluation, we evaluated our model on all known-structure complexes (22 for PROTAC and 96 for molecule glue). Additionally, all similar complexes (with protein similarity bigger than 50%) were excluded from the training data to prevent information leasing. The sample size is determined by taking all available data we have from existing studies. For all the experiments, all the samples were used.
Data exclusions	No data were excluded.
Replication	Results are verified by independent running for five times under different random seeds. Every run includes all of the training samples and tests on the same validation/test set. The performance is stable and reported in supplementary information.
Randomization	We fixed the random seed for better replication. Unless otherwise specified, all training experiments were done by fixing the training random seed to 0. For PROTAC testing, the random seeds are set to 0 - 39.
Blinding	Blinding is not relevant, as this study proposes a deep learning-based method for accelerating PROTAC and molecule glue discovery.

Reporting for specific materials, systems and methods

We require information from authors about some types of materials, experimental systems and methods used in many studies. Here, indicate whether each material, system or method listed is relevant to your study. If you are not sure if a list item applies to your research, read the appropriate section before selecting a response.

Materials & experimental systems

n/a	Involved in the study	
[x]	[ ]	Antibodies
[x]	[ ]	Eukaryotic cell lines
[x]	[ ]	Palaeontology and archaeology
[x]	[ ]	Animals and other organisms
[x]	[ ]	Clinical data
[x]	[ ]	Dual use research of concern
[x]	[ ]	Plants

Methods

n/a	Involved in the study	
[x]	[ ]	ChIP-seq
[x]	[ ]	Flow cytometry
[x]	[ ]	MRI-based neuroimaging

Plants

Seed stocks

Report on the source of all seed stocks or other plant material used. If applicable, state the seed stock centre and catalogue number. If plant specimens were collected from the field, describe the collection location, date and sampling procedures.

Novel plant genotypes

Describe the methods by which all novel plant genotypes were produced. This includes those generated by transgenic approaches, gene editing, chemical/radiation-based mutagenesis and hybridization. For transgenic lines, describe the transformation method, the number of independent lines analyzed and the generation upon which experiments were performed. For gene-edited lines, describe the editor used, the endogenous sequence targeted for editing, the targeting guide RNA sequence (if applicable) and how the editor was applied.

Authentication

Describe any authentication procedures for each seed stock used or novel genotype generated. Describe any experiments used to assess the effect of a mutation and, where applicable, how potential secondary effects (e.g. second site T-DNA insertions, mosiacism, off-target gene editing) were examined.
